# The Effect of Chance Variability in Blood Pressure Readings on the Decision Making of General Practitioners: An Internet-Based Case Vignette Study

**DOI:** 10.1371/journal.pone.0046556

**Published:** 2012-11-02

**Authors:** Mohammed A. Mohammed, Tom Marshall, Paramjit Gill

**Affiliations:** Primary Care Clinical Sciences, University of Birmingham, Edgbaston, Birmingham, United Kingdom; Universidade Federal do Rio de Janeiro, Brazil

## Abstract

**Background:**

Guidelines for the management of blood pressure (BP) in primary care generally suggest that decisions be made on the basis of specific threshold values (e.g. BP 140/90 mmHg); but this fails to adequately accommodate a common cause of variation – the play of chance.

**Objective:**

To determine the impact of chance variability in BP readings on the clinical decision making of general practitioners (GPs) regarding anti-hypertensive treatment and cardiovascular risk management.

**Method:**

We used an internet based study design, where 109 GPs were assigned to manage one of eight case vignettes (guidelines would recommend treatment for only one of the eight) and presented with blood pressure readings that were randomly selected from an underlying population.

**Results:**

Seventeen (15.6%, 17/109) GPs consulted the vignette for whom treatment was recommended, but only 7/17 (41.2%) GPs prescribed treatment, whereas 14/92 (15.2%) GPs prescribed medication to the other vignettes. When deciding to follow-up a vignette GPs were influenced by threshold values for systolic and diastolic BP, but not by the overall cardiovascular risk. If the first reading was a low BP (systolic <140, diastolic <90) GPs were highly likely to discharge the vignette and follow-up a high BP reading (diastolic >90 or systolic BP≥140). Similar factors predicted the decision to prescribe a drug, although the vignette’s cardiovascular risk (>20%) was now statistically significant (p = 0.03).

**Conclusions:**

GP decision making, whilst generally consistent with guidelines, appears to be compromised by chance variation leading to under and over treatment. Interventions to adequately accommodate chance variability into clinical decision making are required.

## Introduction

Like many other countries, guidelines in the United Kingdom (UK) determine whether a patient is recommended antihypertensive treatment based on their measured blood pressure and their ten-year cardiovascular risk [1]Patients are recommended antihypertensive treatment if their ten-year cardiovascular risk exceeds 20% and their blood pressure exceeds 140/90 mm Hg or if their blood pressure exceeds 160/100 mm Hg.

Although guidelines are clear, the decision to start a patient on treatment is not straightforward because within the same individual, measured blood pressure varies. Measured blood pressure exhibits variation of two types: systematic and random (or chance). Systematic variation is caused by a range of patient factors such as flu, the white-coat effect or pain [2] the presence of a medical student or taking blood tests at the time of measurement [3], [4]and by the use of an uncalibrated sphygmomanometer, an inappropriate cuff size, or an insufficient rest period before measurement [5], [6], [7].

The intrinsic biological variability of blood pressure gives rise to “chance like” variation in blood pressure from beat to beat, minute to minute and day to day. To try and take account of blood pressure variability, clinical diagnosis is based on the average of a number of blood pressure measurements: in an attempt to estimate the true, but unknown, mean blood pressure.

Since blood pressure measurement is used as a diagnostic test, variability of blood pressure can lead to false positives – where normotensives misclassified as hypertensive - and false negatives - hypertensives misclassified as normotensive. The Positive Predictive Value [8] (the proportion of test positives that are true positives) of blood pressure measurement is calculable but is known to vary with age [9]. The variability (systematic and chance causes) of blood pressure therefore has the potential to affect clinicians’ decisions to start antihypertensive treatment. Subsequent follow up of patients on treatment also involves blood pressure measurement with systemic and chance variability [10], [11].The effect of this blood pressure variability on clinical decision-making during follow up is also unknown.

Investigating the effect of blood pressure variation on clinical decision-making in clinical practice is challenging because patients with different blood pressures also differ in characteristics such as age, gender or other cardiovascular risk factors and the act of measurement, treatment and management interfere/confound the estimation of the true underlying blood pressure. However simulation, using case vignettes, is one approach to investigating the impact of chance variation on the clinical decision making process because it avoids some of these challenging problems [12]. We used eight typical patient vignettes to study the impact of chance variation in blood pressure on GP clinical decision making.

## Methods

TM sought ethical approval from the South Birmingham Research Ethics Committee and was advised that the project did not require ethical review. All data were handled anonymously.

A website was developed that presented one of eight typical case vignettes of a patient consulting for a routine check up in primary care. The eight case vignettes described a 40, 50, 60 and 70 year old white man and a 40, 50, 60 and 70 year old white woman. In each case the vignette was a non smoking, non diabetic, who drank alcohol in moderation and exercised twice a week, with a body mass index of 25 kg/m2. The total cholesterol level and HDL cholesterol level were the mean for a person of this age and sex based on the Health Survey for England of 1998 [13]. The eight case vignettes are shown in Table 1. Only one vignette, the 70 year old male, would be recommended treatment under UK guidelines. We use the term vignette and patient interchangeably.

**Table 1:** Characteristics of patients in eight case vignettes.

Patientnumber	Gender	Age(years)	Total Cholesterol(mmol/L)	HDL Cholesterol(mmol/L)	Mean blood pressure (95% confidence interval)				Ten-year cardiovascular risk	Whether treatment is recommended
					Systolic (mm Hg)		Diastolic (mm Hg)			
1	Male	40	5.5	1.3	131	(105–157)	77	(67–87)	4%	No
2	Male	50	5.7	1.3	136	(109–163)	80	(70–90)	10%	No
3	Male	60	5.8	1.3	140	(112–168)	81	(71–91)	17%	No
4	Male	70	5.9	1.3	147	(118–176)	82	(72–92)	28%	Yes
5	Female	40	5.2	1.6	123	(99–147)	71	(62–80)	1%	No
6	Female	50	5.6	1.6	130	(104–156)	74	(65–83)	4%	No
7	Female	60	6.1	1.7	138	(111–165)	75	(66–84)	9%	No
8	Female	70	6.4	1.6	147	(118–176)	76	(67–85)	14%	No

* Determined in accordance with UK hypertension guidelines.

Participants who accessed the website were presented with information on the patient’s characteristics, with summaries of current UK guidelines and a hyperlink to an on-line cardiovascular risk calculator [14].Participants were then provided with a blood pressure reading representing the blood pressure measured at a clinic visit. This blood pressure was randomly sampled from a population of two hundred blood pressures with a mean for a person of the appropriate age and gender and a standard deviation reflecting the degree of variation expected within an individual patient from one clinic visit to the next. The standard deviation was based on a coefficient of variation of 9.9% for systolic blood pressure, the variation observed in a meta-analysis of individual patient data from clinical trials of hypertension [15]. Variation in diastolic blood pressure was constrained to show a degree of correlation with systolic blood pressure. This correlation was determined from a series of 59 blood pressures measured in the same individual.

On accessing the website, participants were randomly allocated to one of the eight case vignettes and presented with a first consultation blood pressure reading. Participants were asked to indicate which of three possible decisions they would take. These decisions were coded as 1 to 3 (Table 2). If the participant answer was 1, this was equivalent to discharging the patient, the exercise ended and the participant left the website. If the answer was 2 or 3 the participant proceeded to subsequent consultations (Table 2).

**Table 2:** Possible clinical decisions in the website based case vignettes.

Clinical Decision	
1	Take no action – do not repeat blood pressure measurement in near future
2	Lifestyle advice and/or follow-up – repeat blood pressure measurement in 1 to 3 months
3	Start (or add if already on medication) drug treatment and repeat blood pressure measurement within 1 to 3 months
4	Change one medication and repeat blood pressure measurement within 1 to 3 months
5	Stop one medication.
6	Stop all drugs

At subsequent consultations participants were provided with further randomly selected blood pressure drawn from a population of two hundred. If the patient had been started on treatment the subsequent blood pressure was drawn from a population of two hundred blood pressures with a slightly lower mean, reflecting the effects of treatment. Treatment effects were determined from the average effects reported in meta-analysis [16].The vignettes were adherent to any treatment regimen. Having seen the subsequent blood pressure, participants were asked to indicate their next decision from up to six decisions to account for changes to previous treatment decisions. Participants continued until they discharged the patient or the patient had completed ten clinic visits.

We aimed to recruit UK based GPs, so participants were recruited by emailing members of a number of the Primary Care Cardiovascular Society, the Royal College of General Practitioners and MidRec (the Midlands Research network). Advertisements were placed in Pulse (a UK magazine read by GPs) to draw attention to the study. All participants could opt to be included in a draw with a prize of £100. Participants were asked to provide basic details: their age, gender and clinical specialty.

### Statistical Analysis

Our exploratory analysis involved the use of Classification and Regression Trees (CART) which are a statistical data mining based technique for constructing decision trees by recursively splitting or partitioning patients into homogenous groups [17]. They have been used to support medical decision making [18], [19], [20] although their use is still somewhat novel. Tree models can reflect human decision making and are intuitive to interpret because they have a simple visual presentation, are distribution free, incorporate interaction effects and identify cut-offs for continuous covariates. As first developed, CARTs, could lead to quite large tree models, but recent work has incorporated p-value based tree modelling, known as conditional trees, yielding smaller tree models whilst simultaneously controlling for multiple testing (Bonferroni adjustment, based on p≤0.01) and are available in the *Party* Package [21] in *R* [22]. In the tree models decisions to stop one or all drugs (decision code 5 and 6) were combined and our response variable was the clinical decision (coded as 1 to 5) with the following predictor covariates – vignette no (1 to 8), patients age (years), patients gender (male/female), GPs age (years), GPs gender (male/female), systolic BP (mmHg), diastolic BP (mmHG) and CVD risk (0–100%). We produced a tree model for the first consultation and one for subsequent consultations; although the latter ignores the correlated nature of the measurements (GPs nested within patients) it is nevertheless insightful in highlighting factors which influence a specific decision, if decisions are assumed to be independent.

We used the insights from the tree models to construct two random effects models logistic regression models using the *lme4* [23] package in *R* [22] which reflected the repeated measurements design.

The first model focused on the decision to follow-up (decision 2) the patient and the second model focused on the decision to prescribe medication (decision 3). These two decisions were considered the most important in our study (and other decisions did not occur with enough frequency to enable a meaningful analysis). The covariates in the random effects models were the patients age (years), patients gender (male/female), GPs age (years), GPs gender (male/female), patient’s first visit (yes/no), patients systolic BP≥120 (yes/no), patients diastolic BP≥90 (yes/no) and CVD Risk ≥20% (yes/no). We investigated two way interactions between the blood pressure variables and first visit and retained only those interactions which were statistically significant at the 5% level. GPs were included as random effects as were our eight vignettes (although the latter showed no material contribution – i.e. near zero variance – in the follow-up model.) Our use and choice of threshold values for systolic BP, diastolic BP and CVD Risk was based on values commonly indicated in clinical guidelines (BP 140/90) which were also seen in our exploratory tree models.

## Results

### GP Characteristics

Between September 2007 and July 2008, 109 UK GPs, 65 male and 44 female with an average age of 40.28 years (SD: 9.44), accessed the website and answered questions relating to the case vignettes.

### Decisions at First Consultation

**Figure 1:**
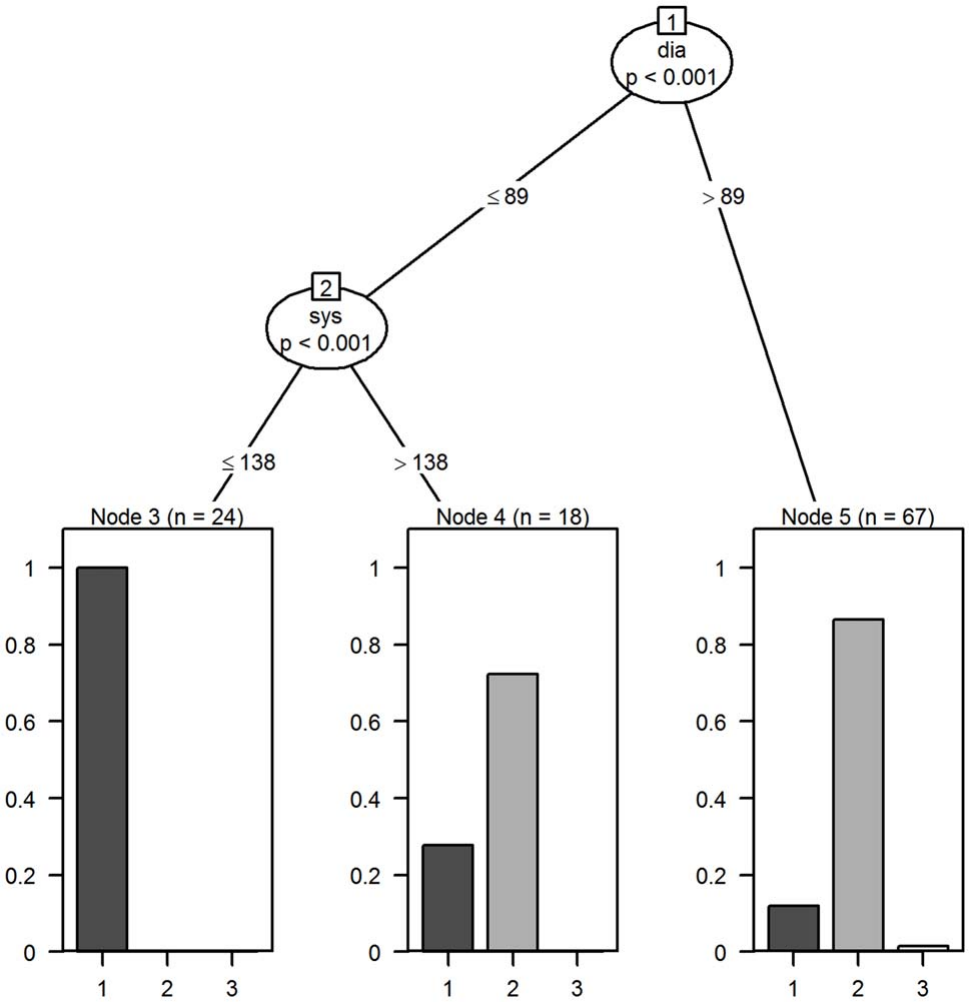
Tree model for decisions made at the first visit for all vignettes. Terminal nodes have proportion on the y-axis and decision code (1 to 3) on the x-axis. Sys is systolic BP and dia is diastolic BP.

Figure 1 shows the conditional tree model for the first consultation involving 109 GPs and the eight vignettes. The model identified three statistically significant (p<0.001) decision pathways which were based on thresholds of systolic and diastolic BP. GP characteristics (age, gender), patient’s ten year CVD risk, patient age, patient gender and patients CVD risk, were not statistically significant features of decision pathways, suggesting that there was no material difference between GPs and that decisions were primarily influenced by threshold values for systolic and diastolic BP.

Specifically if the patient (node1∶2:3, n = 24) had a low BP (systolic ≤138, diastolic ≤89,) then the GPs discharged the patient (n = 24, all discharged). If the patient (node1∶2:4, n = 18) had a low diastolic BP (≤89) but a high systolic BP (>138) then GPs were more likely to invite the patient for a follow-up (n = 18, 13/18 72.2% had a follow-up) and less likely to discharge the patient (5/18 27.7% were discharged). Finally, a high diastolic reading (>89, node 1∶5, n = 67) was most likely to result in a follow-up visit (n = 67, 58/67 86.6% had a follow-up visit), with 8/67 (11.9%) being discharged and 1/67 (1.5%) being prescribed a drug. The decision to prescribe was related to a one-off high BP reading (systolic 175 and diastolic 107) in vignette eight, which equates to ten year CVD risk calculated with this blood pressure reading would be 20.9% (compared to true underlying CVD risk of 13.9%). At the end of the first consultation, 34% (37/109) of GPs had discharged the patient, whereas 65% (71/109) invited the patient for a follow-up consultation and 1 GP had prescribed medication.

### Decisions at Subsequent Consultations

**Figure 2:**
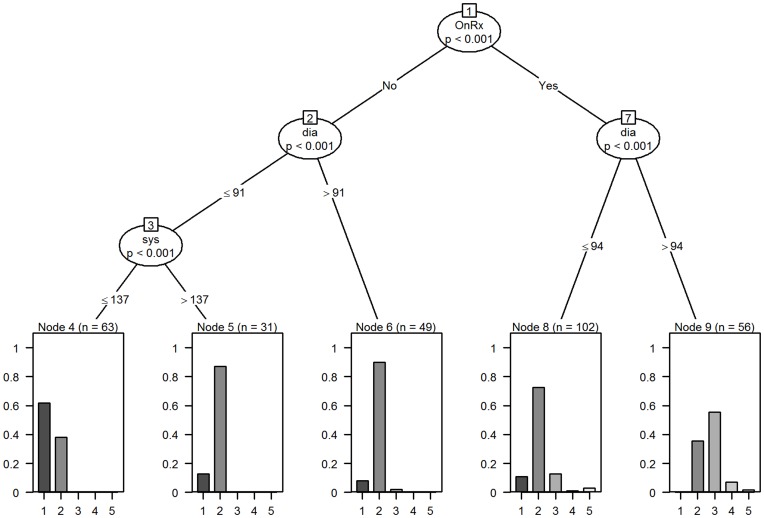
Tree model for decisions made at subsequent visit for all vignettes. Terminal nodes have proportion on the y-axis and decision code (1 to 5) on the x-axis. OnRx stands for on treatment. Sys is systolic BP and dia is diastolic BP.

Figure 2 shows the conditional tree model for the subsequent consultations involving 72 GPs and 301 decisions. This exploratory model identified the patient’s treatment status, the patients systolic and diastolic BP as the key factors driving the GPs decision making process. As in the first consultation, GP factors (age, gender), patient’s ten year CVD risk, patient age and patient gender did not feature. The tree model identified five statistically significant (p<0.001) decision pathways.

If the patient, (node 1∶7:9, n = 56 occasions) was on treatment and presented with a high diastolic BP (>94) then on these occasions, GPs were most likely to add another medication (31/56 additional drug), although in 20/56 such occasions GPs invited the patients for follow-up, on 4/56 such occasions the medication was changed and on one such occasion (1/56) a drug was stopped.

If the patient (node 1∶7:8, n = 102 occasions) was on treatment and presented with a low diastolic BP (≤94) then on these occasions, GPs were most likely to invite the patient for a follow-up (74/102), although in 13/102 occasions GPs prescribed an additional BP drug, on 11/102 occasions GPs discharged the patient, on 3/102 occasions GPs stopped one medication and one occasion (1/102) the GP changed the medication.

If the patient (node 1∶2:6, n = 49) was not on treatment and presented with a high diastolic BP (>91) then on these occasions GPs were most likely to invite the patient for a follow-up (44/49), although on 4/49 occasions GPs discharged the patient and on one occasion (1/49) the GP started the patient on medication.

If the patient (node 1∶2:3, n = 63) was not on treatment and presented with a low diastolic BP (≤91) and a low systolic BP (≤137) then in 39/63 such occasions GPs discharged the patient, whilst in 24/63 occasions GPs invited the patient for follow-up.

If the patient (node 1∶2:3, n = 31) was not on treatment and presented with a low diastolic BP (≤91) and a high systolic BP (>137) then in 27/31 such occasions GPs invited the patient for follow-up, whilst on four (4/31) occasions GPs discharged the patient.

### Treatment Recommendations and Treatment

Table 3 shows the number of GPs who prescribed medication for each of the vignettes. According to UK guidelines, only vignette four (the 70 year old man with high systolic BP – see Table 1) would be recommended treatment and of the 17/109 (15.6%) GPs who consulted this patient vignette, only 7/17 (41.2%) GPs prescribed treatment whereas 14/92 (15.2%) GPs prescribed medication to other patient vignettes.

**Table 3:** GP prescribing patterns for each vignette.

Vignette	Treatment recommendedby guidelines	No of GPs	0 Drug	1 Drug	2 Drugs	3 Drugs	Max no of visitsbefore discharge	Min	Median
1	No	9	9				1	1	2
2	No	18	17	1			4	1	3
3	No	22	16	1	4	1	3	1	3
4	Yes	17	11	3	4		2	1	4
5	No	13	12			1	10	1	3
6	No	13	12	1			9	1	2
7	No	9	6	1	1	1	6	1	3
8	No	8	5		2	1	1	1	3

### Random Effects Statistical Models

#### Decision to follow-up the patient

We further investigated the decision to follow-up the patient using a random effects logistic regression model (Table 4) and found that the GPs decision to follow up a patient was strongly predicted by diastolic BP≥90 or systolic BP≥140, especially at the first visit. If the patient’s BP was below 140/90 then the patient was most unlikely to be invited for follow-up (OR: 0.13). The patient’s ten year CVD Risk≥20%, age, gender, GP age, GP gender were not significant predictors. There was near zero variation between GPs in this respect (SD = 1.41e-7).

**Table 4:** Random effects logistic regression model for decision to follow-up the patient.

Covariate	Odds Ratio	Lower 95%CI	Upper 95% CI	P-value
Patients Age (years)	0.98	0.95	1.00	0.069
Patient Gender(M/F)	1.14	0.69	1.89	0.613
GP Age	1.02	0.99	1.04	0.244
GP Gender(M/F)	0.67	0.41	1.08	0.103
First Visit (yes/no)	0.06	0.02	0.19	<0.0001
Diastolic BP≥90 (yes/no)	1.96	1.05	3.66	0.034
Systolic BP≥140 (yes/no)	3.59	1.57	8.18	0.0024
Risk≥20% (yes/no)	1.39	0.68	2.83	0.368
First Visit * Diastolic BP≥90	25.55	7.00	93.24	<0.0001
First Visit * Systolic BP≥140	7.28	2.05	25.79	0.002
Diastolic BP≥90 *Systolic BP≥140	0.13	0.05	0.36	<0.0001

#### Decision to prescribe a drug

We further investigated the decision to prescribe medication using a random effects logistic regression model (Table 5) and found that the GPs decision to start/add a drug was strongly predicted by whether or not it was the first consultation (OR: 0.02), and diastolic BP≥90, systolic BP≥140 and CVD Risk≥20%. The patient’s age, gender, GP’s age, GPs gender were not significant predictors. There was considerable variation between GPs in this respect (SD = 0.50).

**Table 5:** Random effects logistic regression model for decision to prescribe medication to the patient.

Covariate	Odds Ratio	Lower95%CI	Upper95% CI	P-value
Patients Age (years)	1.05	0.99	1.11	0.132
Patient Gender(M/F)	0.31	0.09	1.06	0.061
GP Age	0.98	0.93	1.02	0.311
GP Gender(M/F)	1.63	0.64	4.17	0.309
First Visit (yes/no)	0.02	0.00	0.21	0.0001
Dia≥90 (yes/no)	8.52	2.95	24.63	<0.0001
Sys≥140 (yes/no)	3.97	1.64	9.61	0.002
Risk≥20%(yes/no)	3.97	1.15	13.74	0.03

## Discussion

To our knowledge this is the first study to explicitly explore how clinician decision making is influenced by chance variation in blood pressure. We found that when deciding to follow-up the patient, GPs appeared not to consider if the vignette’s overall cardiovascular risk was ≥20%, but were influenced by threshold values for systolic and diastolic BP. Furthermore, whilst GPs decisions to prescribe a drug were also affected by similar thresholds, the patient’s cardiovascular risk (>20%) was now seen to be statistically significant. Since cardiovascular risk is only valid in untreated populations we would expect it to influence only the decision to intiate treatment. Finally, we saw evidence of under treatment and over treatment. Under half of practitioners prescribed for the only patient who would be recommended treatment under UK guidelines and about 15% of GPs prescribed medication to non-recommended patients.

Treatment seemed to be determined more by blood pressure than by estimated risk of cardiovascular disease. This observation has also been made in UK clinical practice [24]. Our finding of lower pre-treatment blood pressures in younger patients concurs with that of a survey in New Zealand [25]. It is also consistent with findings relating to cardiovascular prevention through cholesterol lowering: when describing their decision making GPs more commonly mention individual risk factor values than numerical risk estimates [26]. Decision making based on a single risk factor is more compromised by chance variation in that risk factor. Although risk factor variability also affects estimation of global cardiovascular risk. [27].

A weakness of this website based study design is that theoretical decisions are made about a theoretical patient, although we found no evidence of nonsensical decision making, indeed the decisions appeared rational. However as observed earlier, this is the only practical design that can investigate the effects of blood pressure variation on standardised patients. Our participating GPs are self-selected volunteers with an average age of 40.3 years and 59.6% (95% confidence interval: 49.8% to 68.9%) are male. In the UK 52.6% of GPs are male and in England mean age is about 46 [28], [29].The thresholds for systolic and diastolic BP identified by the models are consistent with commonly used clinical guidelines and suggesting that at least in this respect, our GPs are consistent with guidelines.

A key question about the study design is whether the variability of blood pressure in the vignettes adequately represents what is seen in clinical practice. The coefficient of variation of blood pressure used in the case vignettes (9.9%) is consistent with published reports which range from 7.2% to 10% [11], [30], [31].

In the UK it has recently been recommended that 24-hour ambulatory blood pressure measurement be used for diagnosis of hypertension when blood pressures are close to a treatment threshold [32]. Economic modelling has suggested that this may be more cost-effective. [33] However 24-hour ambulatory blood pressure measurements also show variation, with coefficients of variation for mean daily blood pressures (systolic/diastolic) reported as 7.7%/6.6% when measured six months apart and 5.5%/4.9% six weeks apart [34], [35]. But the effects of this new recommendation are complex as clinicians must first decide whether to make use of 24-hour ambulatory blood pressure measurement and then interpret its results. Chance variation will affect both decisions.

Whilst further studies to determine the impact of chance variation on clinical decision making would be useful, it is also worth noting that there is little in the clinical guidelines to mitigate against chance variation and interventions that can help clinicians and patients understand and appropriately react to chance variability are also needed, especially because our findings suggest that addressing chance variability could be a promising strategy for reducing under/over treatment and their associated costs.

## References

[1] National Institute for Clinical Excellence Hypertension Management of hypertension in adults in primary care. (2006) National Institute for Clinical Excellence. Available: www.nice.org.uk/CG018NICEguideline. Accessed 2009 Jul 26.

[2] Reeves RA (1995) Does this patient have hypertension? How to measure blood pressure. Journal of the American Medical Association 273(15): 1211–1218. 7707630 10.1001/jama.273.15.1211

[3] Matthys J, De Meyere M, Mervielde I, Knottnerus JA, Den Hond E, et al (2004) Influence of the presence of doctors-in-training on the blood pressure of patients. A randomised controlled trial in 22 teaching practices. Journal of Human Hypertension 18(11): 769–773. 15141270 10.1038/sj.jhh.1001744

[4] Marshall T, Anantharachagan A, Choudhary K, Chue C, Kaur I (2002) A randomised controlled trial of the effect of anticipation of a blood test on blood pressure. Journal of Human Hypertension 16(9): 621–625. 12214257 10.1038/sj.jhh.1001460

[5] Bakx C, Oerlemans G, van den Hoogen H, van Weel C, Thien T (1997) The influence of cuff size on blood pressure measurement. Journal of Human Hypertension 11(7): 439–445. 9283061 10.1038/sj.jhh.1000470

[6] Rouse A, Marshall T (2001) The extent and implications of sphygmomanometer calibration error in primary care. Journal of Human Hypertension 15(9): 587–592. 11550103 10.1038/sj.jhh.1001241

[7] Bakx JC, Netea RT, van den Hoogen HJM, Oerlemans G, van Dijk R, et al (1999) De invloed van een rustperiode op de bloeddruk. {The influence of a rest period on blood pressure measurement. } Huisarts en Wetenschap 42: 53–6.

[8] Knottnerus JA (2002) The evidence base of clinical diagnosis. London: BMJ Books, 117–43.

[9] Marshall T (2004) When measurements are misleading: modelling the effects of blood pressure misclassification in the English population. British Medical Journal 328: 933. 15087340 10.1136/bmj.328.7445.933 PMC390212

[10] Marshall T (2005) Measuring blood pressure: the importance of understanding variation. Brazilian Journal of Hypertension 12(2): 75–82.

[11] Keenan K, Hayen A, Neal BC, Irwig L (2009) Long term monitoring in patients receiving treatment to lower blood pressure: analysis of data from placebo controlled randomised controlled trial. British Medical Journal 338: b1492. 19406886 10.1136/bmj.b1492 PMC2675695

[12] Peabody JW, Luck J, Glassman P, Dresselhaus TR, Lee M (2000) Comparison of vignettes, standardised patients, and chart abstraction: a prospective validation study of 3 methods for measuring quality. Journal of the American Medical Association 283: 1715–1722. 10755498 10.1001/jama.283.13.1715

[13] Department of Health. (1998) Health survey for England. Available: http://www.data-archive.ac.uk/. Accessed 2005 May 25.

[14] ETHRISK A modified Framingham CHD and CVD risk calculator for British black and minority ethnic groups. Available: http://www.epi.bris.ac.uk/CVDethrisk/CHD_CVD_form.html. Accessed 2009 Jul 27.

[15] Wright JM, Musini VJ (2000) Blood pressure variability: lessons learned from a systematic review. Poster presentation D20, 8th International Cochrane Colloquium. Cape Town. Further details obtained from a personal communication (e-mail) on 21st July 2003

[16] Law MR, Wald NJ, Morris JK, Jordan RE (2003) Value of low dose combination treatment with blood pressure lowering drugs: analysis of 354 randomised trials. British Medical Journal 326: 1427–1432. 12829555 10.1136/bmj.326.7404.1427 PMC162261

[17] Breiman L, Friedman JH, Olshen R A, Stone CJ (1984) Classification and regression trees. Monterey, CA: Wadsworth & Brooks/Cole Advanced Books & Software.

[18] Steyerberg EW (2009) Clinical Prediction Models. A practical approach to development, validation and updating. Springer.

[19] Harper PR (2005) A review and comparison of classification algorithms for medical decision making. Health Policy 71: 315–31. 15694499 10.1016/j.healthpol.2004.05.002

[20] Podgorelec V, Kokol P, Stiglic B, Rozman I (2002) Decision Trees: An Overview and Their Use in Medicine. Journal of Medical Systems, Vol. 26, No.5 10.1023/a:1016409317640 12182209

[21] Hothorn T, Hornik K, Zeileis A (2006) Unbiased Recursive Partitioning: A Conditional Inference Framework. Journal of Computational and Graphical Statistics 15(3): 651–674.

[22] R Development Core Team (2011) R: A language and environment for statistical computing. R Foundation for Statistical Computing, Vienna, Austria. URL http://www.R-project.org

[23] Bates D, Maechler M, Bolker B (2011) lme4: Linear mixed-effects models using S4 classes. R package version 0.999375-40. Available: http://CRAN.R-roject.org/package=lme4.

[24] Mohammed M.A, El Sayed C, Marshall T (2012) Patient and other factors influencing the prescribing of cardiovascular prevention therapy in the general practice setting with and without nurse assessment. Medical Decision Making: In press. 10.1177/0272989X12437246 22357626

[25] Arroll B, Jenkins S, North D, Kearns R (1995) Management of hypertension and the core services guidelines: results from interviews with 100 Auckland general practitioners. New Zealand Medical Journal 108: 55–7. 7885648

[26] Backlund L, Skaner Y, Montgomery H, Bring J, Strender L (2004) GPs’ decisions on drug treatment for patients with high cholesterol values: A think-aloud study. BMC Medical Informatics and Decision Making 4: 23. 15596005 10.1186/1472-6947-4-23 PMC539306

[27] Marshall T (2010) The effect of blood pressure and cholesterol variability on the precision of Framingham cardiovascular risk estimation: a simulation study. Journal of Human Hypertension 24, 631–638. 10.1038/jhh.2009.114 20054346

[28] General Medical Council. List of Registered Medical Practitioners – statistics. Available: http://www.gmc-uk.org/doctors/register/search_stats.asp. Accessed 2012 Aug 21.

[29] Hann M, Sibbald B (2011) General Practitioners’ attitudes towards patients’ health and work. Department for Work and Pensions Research Report No 733. Available: http://research.dwp.gov.uk/asd/asd5/rports2011-2012/rrep733.pdf. Accessed 2012 Aug 21.

[30] Musini VM, Wright JM (2009) Factors Affecting Blood Pressure Variability: Lessons Learned from Two Systematic Reviews of Randomized Controlled Trials. PLoS One 4: e5673. 19479061 10.1371/journal.pone.0005673 PMC2682566

[31] Powers BJ, Olsen MK, Smith VA, Woolson RF, Bosworth HB, et al (2011) Measuring Blood Pressure for Decision Making and Quality Reporting: Where and How Many Measures? Annals of Internal Medicine 154: 781–788. 21690592 10.7326/0003-4819-154-12-201106210-00005

[32] NICE Clinical guideline (2011) 127: hypertension (update). Available: http://www.nice.org.uk/CG127. Accessed 2012 Jan 12.

[33] Lovibond K, Jowett S, Barton P, Caulfield M, Heneghan C, et al (2011) Cost-effectiveness of options for the diagnosis of high blood pressure in primary care: a modelling study. Lancet 378(9798): 1219–30. 21868086 10.1016/S0140-6736(11)61184-7

[34] Brueren MM, van Limpt P, Schouten HJA, de Leeuw PW, van Ree JW (1997) Is a Series of Blood Pressure Measurements by the General Practitioner or the Patient a Reliable Alternative to Ambulatory Blood Pressure Measurement? A Study in General Practice With Reference to Short-term and Long-term Between-Visit Variability. American Journal of Hypertension 10: 879–85. 9270082 10.1016/s0895-7061(97)00125-8

[35] Warren RE, Marshall T, Padfield PL, Chrubasik S (2010) Variability of Office, 24-hour Ambulatory and Self-Monitored Blood Pressure Measurements British Journal of General Practice. 60(578): 675–80. 10.3399/bjgp10X515403 PMC2930221 20849695

